# Residue, dissipation, and dietary intake evaluation of fenpyroximate acaricide in/on guava, orange, and eggplant under open field condition

**DOI:** 10.3389/fnut.2022.939012

**Published:** 2022-08-31

**Authors:** Farag Malhat, Osama Abdallah, Chris Anagnostopoulos, Mohamed Hussien, Indra Purnama, Rania M. A. Helmy, Hanim Soliman, Dalia El-Hefny

**Affiliations:** ^1^Pesticide Residues and Environmental Pollution Department, Central Agricultural Pesticide Laboratory, Agricultural Research Center, Giza, Egypt; ^2^Benaki Phytopathological Institute, Department of Pesticides Control and Phytopharmacy, Laboratory of Pesticides Residues, Athens, Greece; ^3^Department of Chemistry, Faculty of Science, King Khalid University, Abha, Saudi Arabia; ^4^Department of Agrotechnology, Universitas Lancang Kuning, Pekanbaru, Indonesia

**Keywords:** dissipation kinetics, citrus, eggplants, guava, residual behavior, risk assessment

## Abstract

Fenpyroximate is a widely used acaricide applicable in many crops. In this study, the residue behavior of fenpyroximate on eggplant, orange, and guava was investigated. The chronic and acute dietary intake was calculated at several sampling points, and preharvest intervals (PHI) were proposed to ensure compliance with the existing maximum residue levels. A simple extraction protocol combined with ultrahigh-performance liquid chromatography–tandem mass spectrometry (UHPLC-MS/MS) was employed to quantify residue levels. The method was successfully validated according to the European Union (EU) guidelines, and a limit of quantification of 0.01 mg/kg was set. The dissipation patterns in all crops could be described by the first-order kinetics model with half-lives of 1.7, 2.2, and 1.9 days for eggplants, guavas, and oranges, respectively. The dietary risk assessment at the authorized or more critical application patterns was acceptable for the consumers. For oranges and eggplant, a PHI of 3 and 7 days, respectively, can be proposed; however, a proposal was not possible for guava due to the absence of maximum residue limits (MRLs) and quantitative residue findings at all sampling points tested. The current work not only contributes to the practical application of fenpyroximate related to residue management in dryland areas, such as Egypt, but can also be used to estimate the appropriate PHIs and support the authorization of plant protection products as supplementary information.

## Introduction

Pesticides are used to protect crops and increase agricultural yields (1), assuming that they will be applied according to authorized agricultural patterns. On the other hand, the misuse of pesticides may lead to high concentrations of residues in agricultural products, which has forced international agencies and governments to establish maximum residue limits (MRLs) to ensure that safe consumer products enter the market.

**FIGURE 1:**
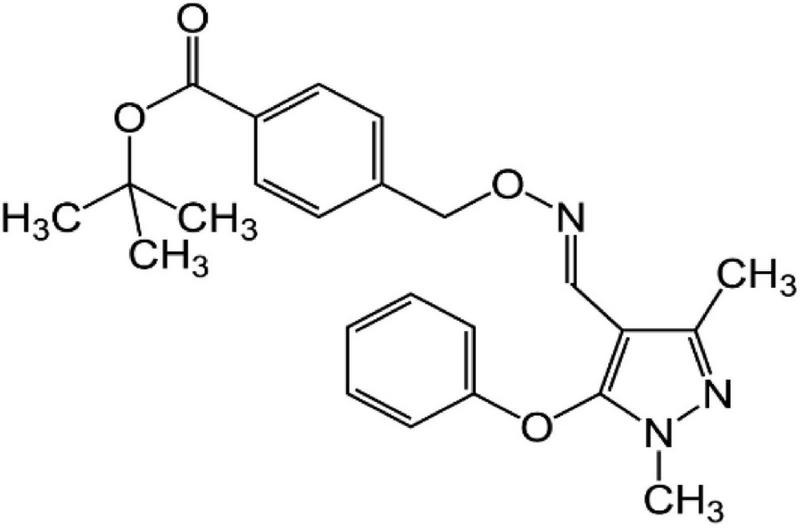
Chemical structure of fenpyroximate (structure was created using ACD/ChemSketch).

One widely used pesticide is fenpyroximate, an acaricide with an oxime-bearing pyrazole structure (Figure 1) developed in 1985 by the Nihon Noyaku Co. (2). It has high efficacy against larvae by inhibiting mitochondrial electron transport (3). Currently, fenpyroximate is widely used for the control of mites in orange, apple, peach, and pear orchards (4). Although fenpiroximate is applicable to many crops, in the current work, the extent of the scope of application was investigated in three additional crops of high economic significance in Egypt: guava, oranges, and eggplants.

Guava (*Psidium guajava L*.) is one of the most consumed edible fruits in tropical and subtropical climates worldwide. In a processed form, it is consumed as beverages, puree, jam, canned slices, syrup concentrate, and juices, with commercial importance in more than 50 countries worldwide (5). Egypt is one of the largest guava producers, with an annual production of 343.703 tons in 2016 (6, 7). In addition to guava, citrus is a major export product of Egypt, which currently exports fruit to the European Union (EU) and the Gulf States. Oranges represent approximately 65% of the Egyptian citrus production, with a total planted area of 162,000 ha (8). Egypt is the sixth-largest producer and the second-largest exporter of oranges globally (9). Eggplant (*Solanum melongena* L.) is a commercial vegetable crop with high demand for most farmers (10). Worldwide, eggplant production has been increasing, with the main producing countries being China, India, Egypt, Turkey, and Japan (11). Eggplant fruits contain a considerable amount of carbohydrates, proteins, and vitamins (12). In Egypt, it is one of the most important crops in the summer season and ranks third worldwide, with an annual production of 2.94% (over 1,180,240 tons) of the total world production (6).

Although field conditions are the ones that affect residue behavior, sensitive and reliable analytical methods are a default requirement for accurate and reliable estimations of residue patterns. Due to the high number of coextracts from the plant matrices, analyte extraction followed by cleanup is required before residue determination (13). The QuEChERS (“quick, easy, cheap, effective, rugged, and safe”) methodology is an extraction protocol first developed by Anastassiades et al. (14) and Lehotay et al. (13, 15, 16). It is commonly used for the extraction of pesticide residues in fruit and vegetables with high water content, replacing conventional extraction techniques (17), which use solvents that generate much hazardous waste or have time-consuming and laborious procedures (18). The coupling of ultrahigh-performance liquid chromatography (UHPLC) with tandem mass spectrometry (MS/MS) detectors is the utmost choice for pesticide residue determination at low levels (19). This combination increased the selectivity and sensitivity of the target analytes and simultaneously reduced the chromatographic run time compared with conventional HPLC techniques (20). However, as the matrix effect (ME) is a common problem for pesticide residue analysis, optimization of the sample preparation step gives more reliable results, minimizing interferences and instrument decay.

In this study, the dietary risk assessment was estimated by taking into consideration the residue levels and dissipation patterns of fenpyroximate in eggplant, orange, and guava cultivations. To ensure reliable measurements, a modified version of the QuEChERS extraction protocol was selected and revalidated according to the EU guidelines (21). Finally, based on the outcome results of the dietary risk assessment and the terminal residue levels found in the products, optimal preharvest intervals (PHIs) were suggested, and compliance with MRLs was checked.

## Experimental

### Chemicals and reagents

Acetonitrile and methanol (HPLC grade) and formic acid (LC–MS grade) were provided by Fisher Scientific Ltd. (Loughborough, United Kingdom). Anhydrous magnesium sulfate (MgSO_4_) (purity, 98%) was purchased from Chem-Lab NV (Zedelgem, Belgium). Extra pure sodium chloride (NaCl) (purity, 99.5%) was acquired from Loba Chemie (Maharashtra, India). The reference standard of fenpyroximate (99.5%, purity) was obtained from Chem Service (West Chester, PA, United States). Fenpyroximate commercial formulation (Ortus^®^, 5% SC, suspension concentrate) (Nihon Nohyaku Co., Ltd., Tokyo, Japan) was secured from a local market. Ultra-pure deionized water was obtained from the Barnstead™ Micro Purification system (Thermo Fisher Scientific, Budapest, Hungary).

### Standard solutions

A stock standard solution of fenpyroximate (1,000 mg/L) was prepared in acetonitrile and stored at –20°C. The intermediate and working solutions were prepared by further dilution in acetonitrile. A solvent calibration curve (standard concentration vs. response) was constructed using acetonitrile, whereas matrix-matched calibration curves were constructed using the extracts from blank guava, orange, and eggplant samples.

### Field experiments, sample collection, and storage

To investigate the residue behavior, two kinds of trials were conducted. Trials (triplicates) estimate the dissipation rate according to the authorized agricultural pattern (1 × 25 g a.i./ha) and trials (triplicates) investigate the terminal residues according to more critical agricultural patterns (2–3 × 25–50 g a.i./ha, 14-day interval).

All trials were carried out under open field conditions during the growing season of 2019/2020. The experimental sites were in El Bhera governorate, an area in mainland Egypt with dry climatic conditions and extensive agricultural activity.

Guava and orange orchards were planted in rows with a row and plant-to-plant distance of 9 m. The experimental field was composed of three replicated plots, with three trees in each plot. To separate the plots with different treatments, a buffer area was maintained between each plot in the trial field. For eggplant, each experimental field consisted of three replicate plots with an area of 40 m^2^ and was separated by irrigation channels.

The temperature of the experimental area ranged between 15 and 27°C during the eggplant, guava, and orange cultivation periods.

Fruit samples from all three plots were collected at 0 (2 h after the last application), 1, 3, 7, 10, 14, and 20 days for the dissipation rate trials and at 3, 7, and 14 days for the terminal residue trials. The size of the sample was at least 2 kg and in line with the guidelines from the Organization for Economic Co-operation and Development (22). Samples were transported to the laboratory, homogenized using a HOBART Food Processor (Hobart Corp., Troy, OH, United States) and stored for a maximum of 1 week in individual polyethylene bags and frozen at –20°C until analysis.

### Pesticide residue analysis

#### Sample preparation and ultrahigh-performance liquid chromatography–tandem mass spectrometry analysis

A modified version of the QuEChERS protocol (23) was used for the extraction. The absorbent portion and the dilution rate were optimized in the current study to minimize the ME.

An aliquot of 10 g of the homogenized sample was weighed into a 50-ml centrifuge tube, to which 10 ml of acetonitrile was added. Then, a piece of a ceramic homogenizer was added, and the tube was manually shaken for 2 min. For the salting out step, a mixture of salts containing 4 g anhydrous magnesium sulfate and 1 g sodium chloride was added. After shaking vigorously for 30 s, the tube was centrifuged for 5 min at 5,000 rpm (ambient temperature). The upper layer extract was filtered through a PTFE 0.22 μm syringe filter (Millipore, Billerica, MA, United States), and then 0.05 ml was transferred into a vial, diluted 20 times using acetonitrile, and vortexed for 30 s before UPLC–MS/MS analysis.

Chromatographic separation and identification of fenpyroximate were achieved using a Dionex Ultimate 3000 RS ultrahigh-performance liquid chromatographic system (Dionex Softron GmbH, Germering, Germany) equipped with a TSQ Altis triple quadrupole mass spectrometer (Thermo Fisher Scientific, Austin, TX, United States). The separation was achieved using an Accucore RP-MS (2.1 × 100 mm, 2.6 μm) C_18_ column (Thermo Fisher Scientific, Vilnius, Lithuania) at a constant temperature of 40°C.

Gradient elution comprised mobile phase A (water containing 0.1% formic acid *v/v*) and mobile phase B (methanol containing 0.1% formic acid *v/v*). The mobile-phase gradient program was 0–1 min 45% B, 1–4 min 90% B, 4–9 min 90% B, 9–9.1 min 45% B, and 9.1–14 min 45% B. A flow rate of 0.3 ml/min and an injection volume of 5 μl were used. Using this program, fenpyroximate was eluted at 8.5 min (SD 0.05%, *n* = 10).

**FIGURE 2:**
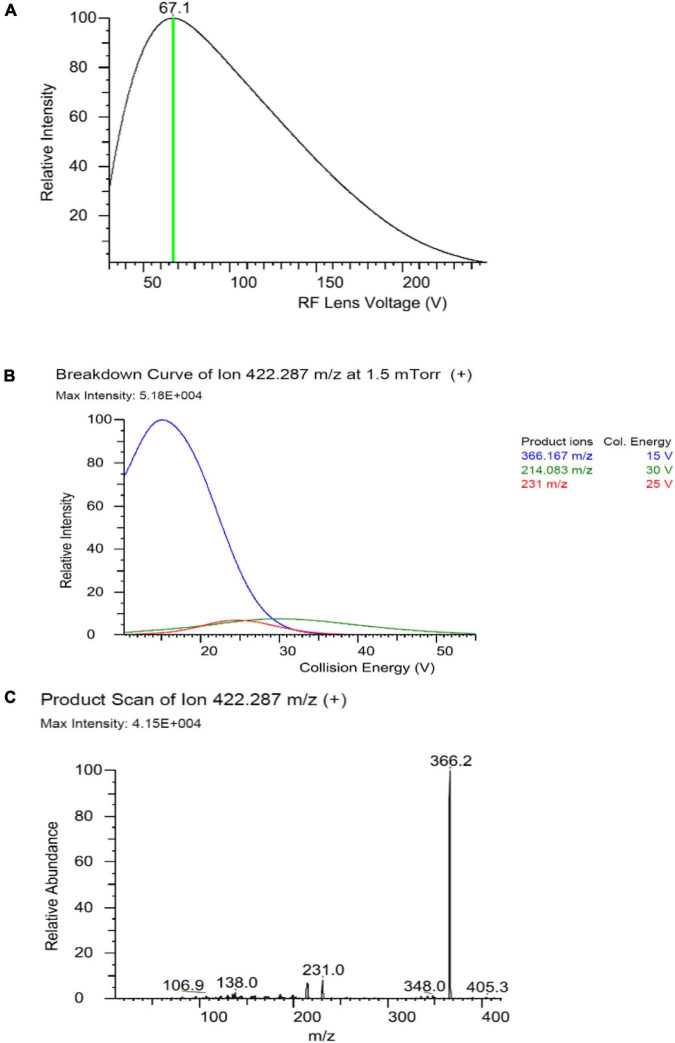
Optimizing Rf Lens voltage **(A)**, breakdown curve **(B)**, and product ion **(C)** of fenpyroximate precursor ion.

The MS/MS analysis was performed in positive electron spray ionization (ESI^+^) in the multiple reaction mode (MRM). For the optimization of the MS/MS conditions (Figure 2), 0.1 mg/L fenpyroximate was infused directly into the system using an infusion pump. The ion transfer tube temperature and vaporization temperature were set at 325 and 350°C, respectively. The capillary voltage was 3,800 V. The auxiliary and sheath gases were set at 10 and 40 bar. The LC–MS/MS parameters are presented in Table 1.

**TABLE 1:** Liquid chromatography–tandem mass spectrometry (LC–MS/MS) parameters for determination of fenpyroximate.

Pesticide	Retention time (min)	Precursor ion (*m/z*)	Product ion (*m/z*)	Collision energy (V)	Rf lens (V)	Dwell time (min)
Fenpyroximate	8.54	422.2	231	24	67	13.3
			366.2	15	67	13.3

#### Method validation

The reliability of the method used was evaluated according to the EU guidelines (21), and the main evaluation criteria were selectivity, linearity, accuracy, limit of quantitation (LOQ), and ME.

Selectivity is the ability of the method to distinguish between the analyte of interest and other molecules present in the matrix (21) and was tested by analyzing blank matrices previously known to be free of fenpyroximate and fortified matrices (*n* = 5, for each matrix) to establish the absence of signals at the elution time of fenpyroximate.

The limit of quantification (LOQ) was estimated as the lower concentration that provides a precision of 80–110% and a precision ≤20%. The default LOQ in the EU is 0.01 mg/kg; thus, this value was selected as the LOQ of the method.

Accuracy was evaluated in terms of trueness and precision. Trueness was studied by determining the recovery percentage. For each matrix, four sets of samples (*n* = 7, for each set) were spiked at levels of 0.01, 0.1, 1, and 4 mg/kg by adding appropriate volumes (not exceeding 200 μl) of fenpyroximate standard solution. Before the extraction step, the spiked samples in the tubes were vortexed for 30 s and allowed to settle for 1 h at room temperature. The spiking levels were chosen to cover the LOQ and concentrations 10, 100, and 400 times higher, thus covering a wide range of concentrations, which are exaggerated for trace analysis, such as residue determination.

Precision was assessed at 0.01 mg/kg in terms of interday repeatability (*n* = 7) and intraday reproducibility (three times with 7-day intervals, *n* = 21).

Linearity was evaluated by constructing calibration curves in acetonitrile and in extracts of each matrix (matrix-matched calibration standards) using ten calibration points between 0.00025 and 0.1 mg/L. In addition, calibration curves were used to estimate the ME by comparing the slopes of the constructed calibration curve in acetonitrile and in matrix-matched calibration standards.

### Dissipation model

The dissipation behaviors and half-lives of fenpyroximate in eggplant, guava, and orange were calculated using the first-order kinetic equations (24); Eqs. 1 and 2:


(1) $$C_{t}=C_{0}\,e^{-k\,t}$$

(2) $$t_{1/2}=l\,n\,2/k$$

where, *C*_*t*_ (mg/kg) is the residue levels of fenpyroximate at time *t* (days), *C*_0_ (mg/kg) is the initial deposits and k is the rate constant (day^–1^).

Microsoft Excel was used for statistical calculations.

### Dietary exposure models

The risks that may result from the long-term intake for the Egyptian consumer were evaluated by using the following equations Eqs. 3 and 4 (24):


(3) N⁢E⁢D⁢I=Σ⁢(S⁢T⁢M⁢R⁢i⁢x⁢F⁢i)/b⁢o⁢d⁢y⁢w⁢e⁢i⁢g⁢h⁢t⁢(b⁢w)

(4) RQ=NEDI/ADI(Eq.4)

where, NEDI is the national estimated daily intake (mg/kg.bw) and STMRi is the median residue data from supervised trials; in our case, since we had three replicates, the mean was used. The ADI, Fi, and bw are the acceptable daily intake (ADI), food consumption data (kg/day), and body weight (kg), respectively. The average body weight is 60 kg for an Egyptian adult (25). Therefore, the risk quotient (RQ) is calculated by dividing the NEDI by the ADI. An RQ value less than 1 represents an acceptable risk for the consumer, while for values higher than 1, the risk is not acceptable. Data were statistically evaluated using one-way analysis of variance (ANOVA), and probability values *p* < 0.5 were considered significant.

In addition, since Europe is one of the main exporters of Egyptian products, to assess the long- and short-term intake for the European consumer, the deterministic EFSA PRIMo revision 3.1 model (26) was employed.

## Results and discussion

### Method development and validation

#### Evaluation of using adsorbent vs. dilution for the cleanup step

The use of adsorbents, such as PSA and GCB in the cleanup step and the effect of dilution at different rates, as a means of reducing the co-extractants in the final extract, were evaluated to minimize the ME, which in the case of LC–MS/MS can be translated into ion suppression in the electrospray source due to competition of the analyte ions with the ions of the coeluting compounds. The ME was considered non-significant if it ranged from –20 to +20%, since this variance value is considered acceptable in terms of repeatability between samples. The effect is considered medium if the value ranges from ±50 to ±20%, whereas the effect is considered strong if the value is below −50% and above +50% (27–29).

The results showed a medium signal suppression effect before the cleanup step. The orange sample extracts showed the highest signal suppression (47.9%) in comparison with eggplant (21.2%) and guava (33.9%) extracts. The addition of PSA at 25 mg/ml of extract or PSA + GCB (25 + 5 mg)/ml of extract had a non-significant influence on the ME of eggplant and guava extracts. In contrast, a significant reduction was observed in the orange extract. When only PSA (25 mg/ml of extract) was used, the ME was reduced from 47.9 to 28.9%, and using the mixture of PSA + GCB reduced it to 12.3%.

**FIGURE 3:**
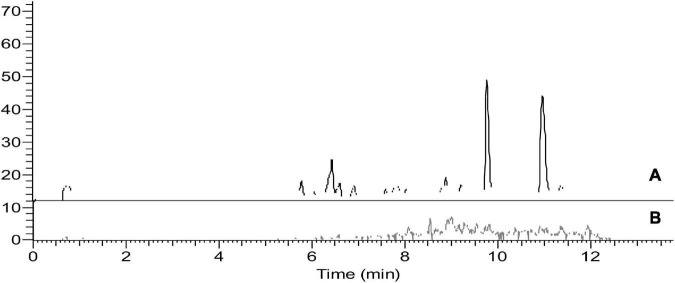
Representative chromatograms of blank orange final extract after 20× dilution. **(A)** Full scan mass spectrum **(B)** Single Ion monitoring of the parent ion 422.2 m/z of fenpyroximate.

**FIGURE 4:**
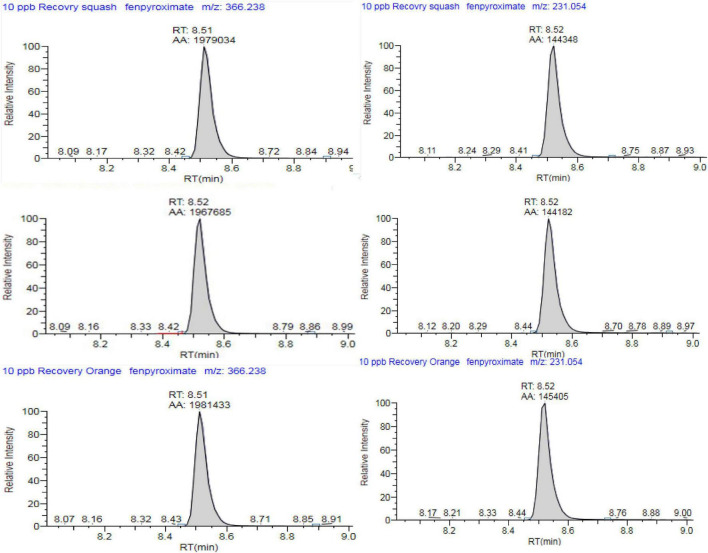
Representative multiple reaction monitoring (MRM) chromatogram of fenpyroximate, TIC for **(A)** m/z = 422.2 to 366.2 and **(B)** m/z = 422.2 to 231 in guava, eggplant, and orange final extract (20× dilution) spiked at 0.01 mg/kg).

To avoid the additional cost of adsorbent materials, extract dilution in solvent was also tested as a means to minimize the ME. In this context, four different dilution factors of 5-, 10-, 15-, and 20-fold for each matrix were tested. In eggplant and guava extracts, the ME was negligible at all tested dilution factors. In orange extract (Figures 3, 4), fenpyroximate shows moderate signal suppression at a dilution factor of 5 and 10, possibly due to the presence of nobiletin and flavonoids in citrus peels, which are considered one of the prominent compounds of poly methoxy flavonoids in citrus fruits (30). Thus, a dilution factor of 20-fold was selected. To have a more robust estimation of the reduction of the ME, the slopes of the calibration curves (acetonitrile and the matrix-matched standards) were compared, *and non-significant (t-test*) matrix suppression effects of –8.3, –2.7, and –5.2% in orange, eggplant, and guava extracts, respectively, (Table 2), were observed.

**TABLE 2:** Linearity, reproducibility, and matrix effect (ME) results from the validation study of fenpyroximate in three commodities.

	Solvent	Eggplant	Guava	Orange
**Linearity**				
Linear range (mg/L)	0.00025–0.1			
Slope	271758	279106	285946	294366
Intercept	–485150	40200	–273498	–349840
*R* ^2^	0.9994	0.9987	0.9994	0.9981
Residual (%)	–16.3	–15.1	–19.8	–14.9
**Reproducibility**				
Intraday repeatability (RSD %) (*n* = 7)^a^	–	7.4	4.4	5.3
Interdays repeatability (RSD %) (*n* = 21)^a^	–	12.8	10.1	15.4
**Matrix effect (ME)**				
% Reduction compared to solvent	–	2.7	5.2	8.3

^a^Evaluated at the LOQ level of 0.01 mg/kg.

**FIGURE 5:**
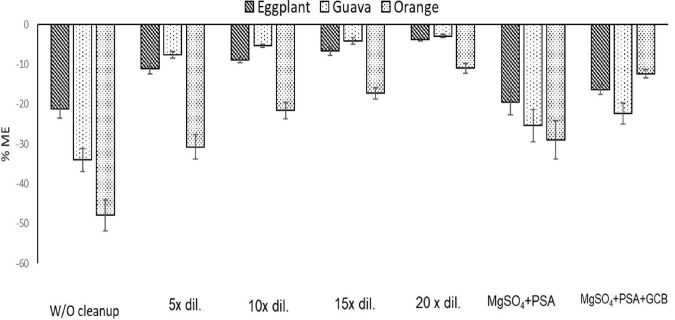
Estimation of the % matrix effect (ME) in eggplant, guava, and orange (*n* = 3) using four dilution and two cleanup, W/O Cleanup:% ME of matrix extracts without cleanup. 5× dilution:% ME of matrix extracts after five times dilution. 10× dilution:% ME of matrix extracts after 10 times dilution. 15× dilution:% ME of matrix extracts after 15 times dilution. 20× dilution:% ME of matrix extracts after 20 times dilution. MgSO4 + PSA:% ME of matrix extracts after cleanup with MgSO4 + PSA. MgSO4 + PSA + GCB:% ME of matrix extracts after cleanup with MgSO4 + PSA + GCB.

A graphical presentation of the impact of adsorbents and sample dilution on the ME of fenpyroximate is presented in Figure 5.

#### Method validation

The selectivity results demonstrated that the matrix co-extractants present in the samples did not give false positives. Regarding linearity, as summarized in Table 2, the results showed a good response for all the tested matrices with a determination coefficient *R*^2^ ≥ 0.998 and residuals ≤19.8% in the range of 0.00025–0.1 mg/L (equivalent to 0.005–2 mg/kg), which shows very good linear regression. The LOQ was set at 0.01 mg/kg in all commodities, with recovery values ranging between 102.3 and 107.3% and relative standard deviation (RSD) ≤ 7.4%. The estimated LOQ value was equal, 50 times lower and 30 times lower than the corresponding lower available MRLs of 0.01, 0.5, and 0.3 μg/kg for guava, orange, and eggplant, respectively.

Regarding precision, values for intraday repeatability were in the range of 4.4–7.4% and for interday repeatability between 10.1 and 15.4%. The higher interday values take into consideration the variability for different days and analysts.

Trueness was evaluated through a recovery study. The obtained recovery values were 93.7–107.3% for eggplant with RSDs of 4.1–8.6% for guava, 92.7–102.3% for eggplant with RSDs of 4.4–7.2% and 92.4–104.3% for orange with RSDs of 3.3–8.5%. The detailed validation results are presented in Table 3. In conclusion, the recovery values obtained for fenpyroximate for all commodities were within the acceptance criterion of 80–110% with a precision RSD of ≤20% (21), meaning the method performed well.

**TABLE 3:** Recovery of fenpyroximate in eggplant, guava, and orange samples (*n* = 7).

Level of fortification (mg/kg)	Eggplant		Guava		Orange	
	% Rec.	%RSD	% Rec.	%RSD	% Rec.	%RSD
0.01	107.3	7.4	102.3	4.4	104.3	5.3
0.1	96.1	4.1	98.6	7.2	95.9	3.3
1	94.5	5.2	97.8	6.7	92.4	8.5
4	93.7	8.6	92.7	6.1	96.8	7.9

### Pesticide residues

#### Dissipation curves and half-life for the studied pesticides

**FIGURE 6:**
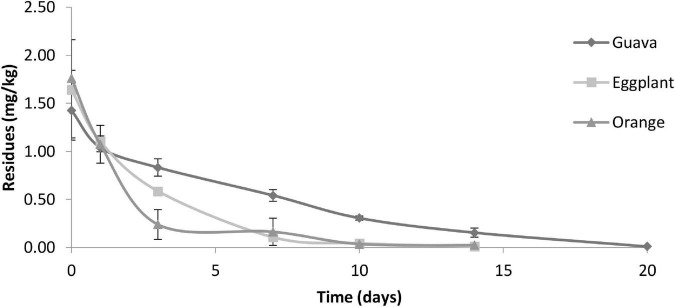
The dissipation behavior of fenpyroximate in eggplant, guava, and orange fruits. The residue concentrations (mg/kg) are expressed in semi-logarithmic scale.

The initial deposits of fenpyroximate in/on eggplant, guava, and orange samples at 0 days (2 h) after application at the authorized dose of 25 g a.i./ha were at 1.64, 1.43, and 1.76 mg/kg, respectively, and exhibited different decreasing tendencies in the tested fruits up to 3 days of application. Approximately 64, 41, and 86% of the initial deposits dissipated, with residue values of 0.58, 0.83, and 0.24 mg/kg, respectively. The decrease was relatively equal up to 7 days after application in eggplant and orange samples. Fenpyroximate showed a lower dissipation rate tendency in guava samples overall to the sampling time compared with eggplant and orange samples (Figure 6). Saku et al. applying fenpyroximate in the authorized agricultural pattern in Egypt, showed that the degradation of 80% was observed after 21 days and 49.5% after 3 days (a half-life estimation was not performed) (31). This is consistent with the residue levels found at a PHI of 21 days but not at a PHI of 3 days.

Despite the similarity of the application rates of fenpyroximate in the tested fruits, there were differences in the initial deposits that are attributed to the crops or the morphological characteristics of each crop or fruit. The outer surface of the orange fruit is rough compared with the smooth outer surface of eggplant and guava, which helps oranges retain a more significant amount of spray solution compared with eggplant and guava.

The dissipation kinetics of fenpyroximate residues in the studied crops are summarized in Table 4. The exponential function was the best descriptor among the other models, and it was found to be *C*_*t*_ = 1.5991e^–0^.^367*t*^, *C*_*t*_ = 1.8051e^–0^.^222*t*^, and *C*_*t*_ = 1.1857e^–0^.^305*t*^ with *R*^2^ values of 0.998, 0.914, and 0.932 for eggplant, guava, and orange, respectively, indicating that the dissipation behavior followed first-order kinetics.

**TABLE 4:** Fenpyroximate decline kinetics in eggplant, guava, and orange fruit.

Days after application	Eggplant	Guava	Orange
	
	Residue (mg/kg)^a^ ± SD (decline%)	Residue (mg/kg) ± SD (decline%)	Residue (mg/kg) ± SD (decline%)
0	1.64 ± 0.52	1.42 ± 0.2	1.76 ± 0.084
1	1.1 ± 0.058 (32.7)	1.04 ± 0.044 (27.1)	1.07 ± 0.197 (38.9)
3	0.58 ± 0.006 (64.2)	0.83 ± 0.090 (41.6)	0.24 ± 0.155 (86.4)
7	0.11 ± 0.023 (93.5)	0.54 ± 0.061 (61.9)	0.16 ± 0.142 (90.7)
10	0.04 ± 0.006 (97.4)	0.31 ± 0.021 (78.5)	0.036 ± 0.002 (97.9)
14	0.011 ± 0.006 (99.4)	0.15 ± 0.047 (89.2)	0.024 ± 0.008 (98.6)
20	BDL^b^	0.01 ± 0.002(99.3)	BDL
Regression equation	*C*_*t*_ = 1.59 e–*^0.362t^*	*C*_*t*_ = 1.79 e^–0.222*t*^	*C*_*t*_ = 1.18 e*^–^*^0.305*t*^
Coefficient(*R*^2^)	0.998	0.914	0.932
Half-life (days)	1.7	2.2	1.9

^a^Average of three replicates.

^b^Below the detection limit.

The half-lives of fenpyroximate were 1.7, 2.2, and 1.9 days in eggplant, guava, and oranges, respectively.

Abd-Alrahman et al. observed almost similar half-lives (*t*_1/2_) of 2.03, 1.56, 2.75, and 2.42 days for apples, grapefruits, grape leaves, and citrus, respectively, when applied at the authorized dose of 25 g a.i/ha (4). Additionally, the half-life of fenpyroximate was 3.5 days in grapes (24). The differences in the half-lives compared with previous studies might be explained by differences in fruit varieties and their masses, growth status, and the morphological structure of plants. In addition, environmental factors may be an explanation for the difference in half-lives and initial residue, such as temperature, sunlight, humidity, microorganisms, soil type, and other factors (32, 33).

#### Terminal residues

The final residual test of fenpyroximate in eggplant, guava, and orange samples was performed at the authorized (25 g a.i./ha) and double (50 g a.i./ha) dose rates at 2 or 3 application rates. Samples were collected after three sampling intervals (3, 7, and 14 days after the last application). A summary of the results is presented in detail in Table 5.

**TABLE 5:** Terminal residues of fenpyroximate in eggplant, guava, and orange fruits.

Dosage (g.a.i/ha)	Number of times sprayed	Days after spraying	Residue (mg/kg)^a^± SD		
			Eggplant	Guava	Orange
25	2	3	0.64 ± 0.01	0.65 ±0.04	1.19 ± 0.08
		7	0.19 ± 0.16	0.16 ± 0.03	0.23 ± 0.06
		14	0.09 ± 0.04	0.03 ± 0.02	0.03 ± 0.01
	3	3	0.86 ± 0.04	0.80 ± 0.06	1.37 ± 0.1
		7	0.32 ± 0.05	0.15 ± 0.03	0.36 ± 0.01
		14	0.04 ± 0.04	0.07 ± 0.03	0.03 ±0.01
50	2	3	0.87 ± 0.1	0.85 ± 0.05	1.28 ± 0.22
		7	0.27 ± 0.05	0.17 ± 0.06	0.81 ±0.12
		14	0.03 ± 0.02	0.03 ±0.01	0.15 ± 0.11
	3	3	1.34 ± 0.2	0.86 ±0.12	2.54 ± 0.49
		7	0.42 ± 0.11	0.21 ± 0.04	0.63 ±0.03
		14	0.04 ± 0.04	0.07 ± 0.01	0.07 ± 0.03

The initial residues in eggplant and guava were similar (0.64–1.34 mg/kg in eggplant and 0.65–0.86 mg/kg in guava) and slightly lower than those in oranges (1.19–2.54 mg/kg). In all cases, the values of fenpyroximate residues decreased with the sampling interval, with a total degradation of 86–98% after 14 days.

By applying the authorized dose rate at 2 or 3 applications, residues ranged from 0.04 to 0.86 mg/kg, 0.07 to 0.8 mg/kg, and 0.03 to 1.37 mg/kg in eggplant, guava, and orange, respectively. The degradation of fenpyroximate between the 7th and 14th day after the application was 10–24% in all cases except in eggplant when the application was performed 3 times, in which the degradation was 33%. This difference is related to the fact that a lower degradation (63%) was observed after 7 days.

In the worst-case application pattern of double the dose rate and 2 or 3 applications, residues ranged from 0.04 to 1.34 mg/kg, 0.07 to 0.86 mg/kg, and 0.07 to 2.54 mg/kg at eggplant, guava, and orange, respectively. Similar to the previous trials, the degradation of fenpyroximate between the 7th and 14th day after the application was 16–28%; the exception, in this case, was in oranges when the application was performed two times, in which the degradation was 58%. Additionally, in this case, the difference is related to the fact that a lower degradation (63%) was observed after 7 days.

Overall, a similarity in the degradation of fenpyroximate was observed in all three crops. Terminal residues at 7 days after the last application were degraded compared with 3 days by 31–37% in eggplant, 19–25% in guava, and 19–26% in oranges (except in one case where the degradation was up to 63%). Similarly, terminal residues at 14 days after the last application were degraded by 3–14%. Based on the results, neither the number of applications nor the dose rate affected the degradation pattern.

#### Consumer risk assessment

For the exposure calculations, as input values, the mean (three replicates) of fenpyroximate in eggplant, guava, and orange were used, except in the cases where residues were below the LOQ, for which the value of 0.01 mg/kg was used. The results were compared with the ADI of 0.01 mg kg^–1^ bw/day in the case of long-term intake and with the acute reference dose (ARfD) of 0.02 mg kg^–1^ bw/day in the case of short-term intake (34).

For Egyptian consumers, following the RQ approach, the long-term exposure ranged from 5.24E^–05^–5.18E^–03^ mg/kg bw for eggplant, 1.53E^–05^–9.94E^–04^ mg/kg bw for guava, and 3.52E^–05^–4.14E^–03^ mg/kg bw for orange. The corresponding RQs ranged from 0.01–0.52, 0.002–0.1, and 0.004–0.41, respectively. The results are summarized in Table 6. The dietary risk levels were found to be less than 1. Therefore, if fenpyroximate is applied according to the authorized and more critical application patterns and the fruits are harvested after the sampling times of 3, 7, and 14 days, the risks for the Egyptian consumer due to the long-term dietary intake are low, and the exposure for the consumer is acceptable.

**TABLE 6:** Long-term exposure calculations of fenpyroximate in eggplant, guava, and orange fruits for the Egyptian consumer using the RQ approach.

Dosage (g a.i/ha)	Number of times sprayed	Days after spraying	Eggplant			Guava			Orange		
			Mean	NEDI	RQ	Mean	NEDI	RQ	Mean	NEDI	RQ
25	2	3	0.64	2.30E-03	0.23	0.65	7.07E-04	0.07	1.19	1.82E-03	0.18
		7	0.19	7.04E-04	0.07	0.16	1.73E-04	0.02	0.23	3.54E-04	0.04
		14	0.09	3.37E-04	0.03	0.03	3.28E-05	0.00	0.03	4.58E-05	0.00
	3	3	0.86	3.23E-03	0.32	0.8	8.63E-04	0.09	1.4	2.10E-03	0.21
		7	0.32	1.20E-03	0.12	0.15	1.65E-04	0.02	0.36	5.47E-04	0.05
		14	0.04	1.50E-04	0.01	0.07	7.57E-05	0.01	0.03	4.59E-05	0.00
50	2	3	0.87	3.25E-03	0.33	0.85	9.15E-04	0.09	1.3	1.96E-03	0.20
		7	0.27	1.03E-03	0.10	0.17	1.83E-04	0.02	0.81	1.24E-03	0.12
		14	0.03	1.12E-04	0.01	0.03	3.23E-05	0.00	0.15	2.30E-04	0.02
	3	3	1.3	5.02E-06	0.00	0.86	9.29E-04	0.09	2.5	3.88E-03	0.39
		7	0.42	1.56E-03	0.16	0.21	2.29E-04	0.02	0.63	9.68E-04	0.10
		14	0.04	1.50E-04	0.01	0.07	7.53E-05	0.01	0.07	1.07E-04	0.01

NEDI, national estimated daily intake; RQ, risk quotient.

As for the European consumer, as input data, the mean residue concentration measured from all agricultural patterns and all PHIs was applied. In the case of oranges, a peeling factor of 0.24 was applied (35). The long-term exposure was calculated to be up to 5% of the ADI for eggplant, 0.5% for guavas, and 25% for oranges; thus, a chronic risk to the consumer is not observed. The short-term exposure was calculated to be up to 222% of the ARfD for eggplant, 157% for guava, and 404% of the ARfD for oranges. The results are summarized in Table 7.

**TABLE 7:** Long-term and short-term exposure calculations of fenpyroximate in eggplant, guava, and orange fruits for the European consumer using the EFSA PRIMo revision 3.

Dosage (g a.i/ha)	Number of times sprayed	Days after spraying	Eggplant			Guava			Orange		
			Mean	Maximum ADI (%)	Maximum ARfD^a^ (%)	Mean	Maximum ADI (%)	Maximum ARfD^a^ (%)	Mean	Maximum ADI (%)	Maximum ARfD^a^ (%)
25	1	0	1.64	5	**222**	1.42	0.5	**157**	1.76	17	**280**
		1	1.1	4	**149**	1.04	0.4	**115**	1.07	10	**170**
		3	0.58	2	73	0.83	0.3	92	0.24	2	38
		7	0.11	0.4	14	0.54	0.2	60	0.16	2	25
		10	0.04	0.1	5	0.31	0.1	34	0.036	0.3	6
		14	0.011	0	1	0.15	0.1	17	0.024	0.2	4
		20	< 0.01	0	1	0.01	0	1	<0.01	0.1	2
25	2	3	0.64	2.00	87.00	0.65	0.20	72.00	1.19	11.00	**189.00**
		7	0.19	0.60	36.00	0.16	0.1000	18.00	0.23	2.00	37.00
		14	0.09	0.30	12.00	0.03	0.00	3.00	0.03	0.30	5.00
	3	3	0.86	3.00	**116.00**	0.80	0.30	88.00	1.37	13.00	**218.00**
		7	0.32	1.00	43.00	0.15	0.10	17.00	0.36	3.00	57.00
		14	0.04	0.10	5.00	0.07	0.00	8.00	0.03	0.30	5.00
50	2	3	0.87	3.00	**118.00**	0.85	0.30	94.00	1.28	12.00	**204.00**
		7	0.27	0.90	37.00	0.17	0.10	19.00	0.81	8.00	**129.00**
		14	0.03	0.10	4.00	0.03	0.00	3.00	0.15	1.00	24.00
	3	3	1.34	4.00	**181.00**	0.86	0.30	95.00	2.54	25.00	**404.00**
		7	0.42	1.00	57.00	0.21	0.10	23.00	0.63	6.00	**100.00**
		14	0.04	0.10	5.00	0.07	0.00	8.00	0.07	0.70	11.00

^a^Bold are the values where an exceedance of the ARfD is observed. ADI, acceptable daily intake; ARfD: acute reference dose.

In eggplant, for residues resulting from the agricultural patterns of 1 × 25 g a.i./ha, 0–1 day PHI; 3 × 25 g a.i./ha, 3 days PHI, and 2–3 × 50 g a.i./ha, 3 days PHI, an exceedance of the ARfD was observed. Similar to guava, for residues resulting from the agricultural patterns of 1 × 25 g a.i./ha, 0–1 day PHI. In oranges, the same applies to the agricultural patterns of 1 × 25 g a.i./ha, 0–1 day PHI; 2–3 × 25 g a.i./ha, 3 days PHI and 2–3 × 50 g a.i./ha, 3 and 7 days PHI. Thus, these agricultural practices should not be used, and the information will be taken into consideration in the discussion of the proposal of the PHI.

#### Maximum residue limits and preharvest intervals

For fenpyroximate, the codex MRL in citrus fruits was set at 0.6 mg/kg, in eggplants at 0.3 mg/kg, and no value was set for guava. In the EU legislation, the EU MRLs (36) are set at 0.5 and 0.3 mg/kg for citrus fruits and eggplants, respectively, whereas for guava, the MRL is set at the LOQ of 0.01 mg/kg. In Egypt, national MRLs are not set; thus, the codex MRLs apply to ensure consumer safety. Based on the residue levels observed per agricultural pattern and the outcome of the consumer risk assessment, the following PHIs are proposed:

For eggplant, a minimum PHI of 7 days is proposed if fenpyroximate is applied according to the authorized application pattern. In the case where the application is conducted with a more critical pattern, the PHI varies between 7 and 14 days depending on the residue levels. When fenpyroximate is applied at 2 × 25 or 50 g a.i./ha, a PHI of 7 days is proposed, and when fenpyroximate is applied at 3 × 25 or 50 g a.i./ha, a PHI of 14 days is proposed.

For guava, at all sampling points, quantitative residues were found. Since the EU MRL is set at the LOQ of 0.01 mg/kg, a PHI cannot be proposed, neither when fenpyroximate is applied according to the authorized nor a more critical pattern. However, after 20 days of the last application, when fenpyroximate was applied according to the authorized pattern, residues were at the LOQ (0.01 mg/kg), and according to the dissipation pattern in guava, residues below the LOQ were expected to be present after 25 days.

For oranges, a minimum PHI of 3 days is proposed if fenpyroximate is applied according to the authorized application pattern (1 × 25 g a.i./ha). In the case where the application is conducted with a more critical pattern, a PHI of 7 days is proposed if applied 2–3 times at the authorized dose rate (25 g a.i./ha) and 14 days if applied 2–3 times double the authorized dose rate (50 g a.i./ha).

## Conclusion

An easy and effective approach using a modified QuEChERS pretreatment, with an additional 20-fold dilution step to minimize the MEs and combined with UPLC–MS/MS, was validated for the determination of fenpyroximate in eggplants, guavas, and oranges. The dissipation patterns in all crops could be described by the first-order kinetics model with half-lives of 1.7, 2.2, and 1.9 days for eggplants, guavas, and oranges, respectively. Additionally, the dietary risk assessment at the authorized or more critical application patterns was performed for Egyptian and European consumers, where exceedance of the ARfD were observed for residues at 0 and 1 day PHI for the authorized agricultural practices and at 3 and 7 days PHI for some more critical agricultural practices. In addition, in all cases, residues were not below the existing MRLs. Thus, setting a PHI is essential to avoid exceedances that have trade restrictions as a consequence or to ensure consumer safety. For oranges and eggplant, a PHI of 3 and 7 days, respectively, can be proposed if fenpyroximate is applied according to the authorized application pattern. For guava, due to the absence of MRLs and since quantitative levels were found in all cases, an accurate PHI cannot be proposed; however, based on the dissipation pattern, quantitative residues after 25 days are not expected.

The current work not only contributes to the practical application of fenpyroximate related to residue management in dryland areas, such as Egypt, but can also be used to estimate the appropriate PHIs and can support the authorization of plant protection products as supplementary information.

## Data availability statement

The original contributions presented in the study are included in the article/supplementary material, further inquiries can be directed to the corresponding author/s.

## Author contributions

FM: designed and coordinated the study, developed analytical method, analyze the samples, interpret the results, edited, and wrote the manuscript. CA: wrote the manuscripts, bibliography search, performed statistical analysis, interpretation of the results, and perform the risk assessment. OA and MH: conducted the experimental design, field trial, analysis of the samples. RH, DE-H, and HS: conducted the experimental design, field trial, and analysis of the samples. All authors read and approved the final manuscript.
